# Headaches and Neuralgias Due to Diseases of the Nose and Accessory Sinuses

**Published:** 1910-11

**Authors:** Hugh M. Lokey

**Affiliations:** Atlanta, Ga.


### HEADACHES AND NEURALGIAS DUE TO DISEASES OF THE NOSE AND ACCESSORY SINUSES.

By Hugh M. Lokey, M. D., Atlanta, Ga.

The subject of my paper is not a new one. But there is so little written in text-books on this, most of literature on the subject being in the nose and throat journals, and in reprints, which seldom reach the man in general practice, and as I consider this a very important subject for the consideration of all branches of the profession—headaches being a trouble we all have to treat—I hope to interest you in this paper.

First, let us take up the anatomy of the nose and accessory sinuses, briefly considering the large area of mucous membrane involved, and the blood and nerve supply. It will be easier then to understand how reflex irritation and pain, distant from the area of inflammation, can be produced.

The nasal cavities are separated from each other by the nasal septum, which forms a perpendicular inner wall to each. The outer borders are formed by the lâteral nasal walls, from which project the turbinate bodies dividing the meati.

The sinuses we will here consider are the maxillary, on Antrum and Highmore, situated one in the body of each superior maxillary bone, lying to the outer side of the nasal cavity, and beneath the orbital cavities; the frontal sinuses situated in the frontal bone, above the nasal and the orbital cavities; the ethoidal sinuses, or cells, in the ethmoid bone, lying between the nasal and orbital cavities, and the sphneoidal cells in the body of the sphenoid bone, at the upper and posterior part of the nasal cavity.

The mucous membrane lining the nasal cavity and all the sinuses is continuous, that over the turbinate bodies having a thick underlying submucous membrane in which there are a large number of veins. The blood supply of the nasal fossa is from the sphenopalatine artery, a branch of the internal maxillary. Two branches are given off from the sphenopalatine, the internal going to the septum and the external to the lateral walls, ethmoid cells, frontal sinus and antrum of Highmore. The anterior and posterior ethmoidal arteries supply short branches to the attic, ethmoid cells and frontal sinuses. The veins empty into the pharyngeal plexus, and the intracraneal vein. The nerve supply to the septum, the nasal walls, turbinates and accessory sinuses is chiefly from sensory branches of the fifth nerve, which anastomose with branches from the sympathetic. It is to the extensive distribution of the fifth nerve that the varied forms of headaches, neuralgias, migraines, ticdouloureux, etc., are due.

This nerve distributes sensory fibers to the dura, pia, orbit, eyelids, nose, gums, teeth, tonsils, palate, sphenoid and ethmoid cells, frontal and maxillary sinuses, nasal fossa, pharynx, ear, articulation of lower jaw, parotid gland, scalp, forehead and face. Pressure upon filaments in one branch may be referred back through the gasserian gangloin to the extremity of another branch. It has been a common practice for the physician to prescribe anodynes and opiates for facial neuralgias, and for the dentist to extract teeth that were sound, in hope of relieving the intense pain. This leads me to urge that you first seek the cause, and remedy it, giving your patient permanent, instead of temporary, relief, and shield him from dangerous complications, placing him on the basis with the patient with appendicitis, and not the one with cramp colic.

The acute inflammations are most frequently due to an attack of grippe, when frequently the entire mucous membrane is engorged. The patient may have no temperature, or the temperature may rise to 103 or 104.

The symptoms in these cases of nasal headaches are such that it is frequently very difficult to determine the exact location of the morbid condition.

If, on examination you find that a septal spur, or deviation, impinging upon the lateral wall or one of the turbinates pressed tightly against the septum or the outer nasal wall, the patient complaining of a tight feeling in the nose, pain across the bridge of the nose, in the eyes, and radiating over the forehead and temples, and possibly more in the supraorbital region than elsewhere, your diagnosis is easily made. But, if the trouble lies in one of the sinuses, it will be more difficult, in fact, impossible, to be exact. In the acute stage of sinusitis, before there is a discharge from the sinus, we have to rely very much upon the location of the pain. The pain is more severe in the chronic stage, but may be less so at the diseased point than elsewhere.

In acute suppuration of the antrum, the teeth on the affected side may ache, usually the second bicuspid or the first molar. There will be soreness over the maxilla, and infraorbital pain may be complained of, and in some cases supraorbital pain will be present.

In acute frontal sinusitis, there will be pain over the frontal bone, and over the inner portion of the eyebrow, which may be referred to the ear, or the side of the head. Patients also complain of pain when blowing the nose. Pain from ethmoiditis is felt in the lower portion of the forehead between and back of the eyes.

Dr. Sargent F. Snow suggests that "a pain shooting from the bridge of the nose outward and upward indicates an envolvement of the anterior ethmoid. A deeper and more intense pain under or behind the eyes, and sometimes apparently in the ear or temple, points to the middle and posterior ethmoid, while a deeper, splitting pain, radiating outward from the center, sometimes reflects around the anterior third of the lower jaw, is relieved by opening the sphenoid. Accumulations within the maxillary antrum are diagnosticated by the localized pain."

Dr. W. Campbell Posey suggests that neuralgia of the supraorbital nerve is very significant of frontal sinus disease; frontal headache is suggestive of ethmoidal and frontal disease; occipital is usually caused by ethmoiditis, though disease of the frontal sinus may evidence itself in pain in that locality also.

I saw a case some time ago, referred to me by a dentist. She consulted him for pain in the left upper teeth and her antrum. He could find no cause for pain. I found the left midtle turbinate, though only moderately engorged, pressed tightly against the septum. For diagnostic purposes, and let me say here that only for diagnostic and operative purposes do I use cocaine, I applied a 4% sol. of cocaine and a 1 to 8,000 sol. of adrenalin to the swollen turbinate, and as soon as it was sufficiently depleted to be free from the pressure, the pain stopped, though, it required several weeks' treatment to effect a cure.

I have seen the pain from acute anterior ethmoiditis more severe in the orbit and supraorbital region than in the immediate area of the disease, though there would be a feeling of tightness and pain in the nose, and pain could be produced by pressing the nose at the inner angle of the orbit. I saw a case recently in which the pain at first was more severe in the orbit, across the bridge of the nose and the supraorbital region, and in a few days developed an empyemia of the antrum. This might be due to the location of the ostium maxilla, being so situated that frequently the fluid discharged from the frontal sinus or anterior ethmoid cell drains into it, and is retained in the antrum. But in this case there was no discharge from either of these sources, and transillumination showed an engorged condition of the sinus, even after it was emptied of the secretions, and pressure over the antrum was painful, showing this to be the seat of the inflammation.

In chronic sinusitis the pain is less severe unless polyps, or extensive granulations are present, and the secretions are retained, producing pressure.

For ease of diagnosing, we will divide the sinuses into anterior and posterior. The anterior, the antrum, the frontal sinus, and the anterior ethmoid cells, all of which empty beneath the middle turbinate into the hiatus semilunaris, which forms part of the middle meatus; and the poserior, the middle and posterior ethmoids, and the sphenoidal cells, which enter into the superior-meatus. If pus is discharging from the sinus affected, you can localize it by the location of your discharge. That from the frontal sinus dripping down from over and around the anterior end of the middle turbinate, and that from the ethmoid and antrum coming from further back, and, if it is from the antrum, it can be increased by having the patient lean forward and turn the head, with the affected side up.

Discharge from the middle and posterior ethmoid cells will run down over the posterior portion of the middle turbinate, and from the sphenoidal sinus most of the discharge will drip in the throat. Transillumination is a great aid in diagnosing an empyema of the antrum, ethmoid cells or frontal sinus, and if the disease is far enough advanced, pressure over the area will produce pain. When we bear in mind the nerve supply of these parts, we can readily understand, if the same nerve reflex exists here that do in other parts of the body, why these remote and radiating pains are produced.

These conditions can, in a great many acute and some chronic cases, be relieved by treatment, but a large per cent., especially in chronic cases, are operative. No one should jump at conclusions, and some of these cases can only be diagnosed by exclusion. Unless positive evidence is present of morbid conditions here, a thorough examination of the eyes should be made, and other functional and organic conditions investigated. As to the relief of these conditions, I will not go into the more complicated means, but simply mention minor operations, as it will not be possible to describe all operative methods for lack of time.

If there be an exostosis, or spur, of the septum pressing on the outer nasal wall that the pressure pain is attributed to, it should be done with a saw or a dental burr. I prefer the latter, as it can be done with neater surgical tecnique. I have done a number of these by the submucous flap method (dissecting up the mucous membrane and periostium and burring off the spur, replacing the flap and dressing). After healing there is much less cicatricial tissue left. I have tried this on removed of the turbinate, but find it more difficult than would be imagined.

If pressure is caused by a turbinate, either inferior or middle, pressing against the septum or lateral wall, or simply in an engorged or hypertrophied state, possibly making no pressure at any one point, but still producing a pressure upon the delicate nerve fibres within itself, due to the turgescent circulation, or hyperplasia, it should be reduced by daily applications of 1 to 8,000 adrenalin solution, and stimulating applications of camphor menthol, if in the acute stage; but, if hyperplastic or of a cystic nature, it should be punctured by the actual cautery or removed with scissors of snare. In the acute conditions, local treatment usually relieves the patient, but if of a chronic nature, the greater per cent. is operative.

If polyps exist (though easily located usually, sometimes can only be seen by the closest examination, lying in the recesses behind the turbinates), they should be removed with snare and forceps.

One author reports a case of headache relieved by removal of adenoids. Of course, if these are present they should be removed with currette or forceps.

We now come to the more difficult problem: Diagnosis, and treatment of the sinus diseases. Bear in mind that nature, free drainage, cleanliness and free areation of these will frequently effect a cure. As stated above, I will not go into the radical operations of the sinuses, a thing seldom required in this climate.

Possibly the most frequently diseased sinus is the maxillary, being closely related to the alveolar processes, where diseased teeth will affect, and with its source of drainage, the ostium, at the upper portion of the wall, mucous or pus is naturally retained in the cavity.

There are several methods of diagnosing and treating diseases of this cavity. Tenderness and pressure over the antrum, and pain, infraorbital, and across the nose, and sensitiveness of the bicuspid, or first molar tooth, indicate antral trouble. Transillumination with a complete shadow of the alveolar margin, is a good, though not positive evidence. The removal of the second bicuspid, or first molar tooth, and drilling with a dental burr into the caity will be a source of positive diagonsis, and will also serve as an avenue of drainage, or by puncturing the lateral walls beneath the anterior end of the inferior turbinate about one inch from the meatus with the canular trochar and irrigating with normal saline solution, you can confirm your diagnosis, and also relieve the cavity of retained secretions. I prefer the latter. If pus is found, and several irrigations in the manner do not relieve, an opening should be made with a Myles punch large enough to afford constant drainage. This can be better accomplished by the removal of the anterior one-third of the inferior turbinate. Where accumulations of polyps exist within, the more radical procedures are required, opening and curretting.

The frontal sinus has the best natural drainage of all the accessory sinuses, but if there be granulations or polyps about the opening of the naso frontal duct, either at the nasal or sinus end, or if the middle turbinate is engorged or hypertrophic, you have a damming up of the secretions and consequent pain. The simplest relief to these cases, is to remove with the snare, scissors, or forceps, the anterior end of the middle turbinate, and if granulations or polyps are present in the hiatus semilunaris, to remove them with the currette and forceps, and unless you have an advanced case, the free drainage thus secured is in a large degree all of the operating necessary.

The anterior ethmoidal cells are frequently involved, and are relieved by the same operative procedure. However, if granulations or polyps exist in these cells, they should be broken down and curretted out.

*Discussions on the paper of Dr. H. M. Lokey, Atlanta, Ga.—*

Dr. B. F. Rae, LaFayette, Ala.—I can add nothing to the Doctor's paper. Consider it an excellent paper. There is, however, one point of interest I may give just here, that is, the antiseptic spray for acute colds or headaches. I consider quinine best, as I have found it a great thing for Grippe and headaches: I think it will abort a case of grippe and do much good.

Dr. J. L. Campbell, Atlanta, Ga.—I have little to say on this paper except, that it is of considerable importance. It deals with a subject that the general practitioner should know more about, or advise the patient to go to some one who does know something about it.

Dr. H. M. Cole, Mobile Ala.—I enjoyed the Doctor's paper; it is a very interesting subject, but is entirely over my head, as I have had very little experience along that line. Here, however, Dr. Cole related a very interesting case of a gentleman 64 years of age, on whom he had operated in the past few weeks.

Dr. J. G. Palmer, Opelika, Ala.—I did not hear all of the Doctor's paper, however, from what I did hear, I judge it to be a fine paper, a very striking paper. It calls to my mind a conversation I had with a gentleman, a man of more than ordinary intelligence, six or eight months ago. His health became impaired, and it was reported that he was dying with Bright's Disease, in fact, his case had been diagnosed as such. I saw him last Sunday, and he was in the best of health, although he had been told that he would die. He had suffered a great deal with two of his teeth, and had insisted on having them pulled, and finally, against the advice of both physician and dentist, he did have them extracted.

Gradually the disordered stomach, and other symptoms of malaria, as well as the symptoms of Bright's Disease passed away. It tends to show how often we are misled in our diagnosis.

Dr. Lokey, in closing.—I thank the gentlemen for their discussion, do not know that I have anything particular to add. Want to say to Dr. Rea that the quinine is good for a spray, or just to snuff in the nose. Remember some time ago that some one told me of this remedy, and I remember their measure was, to take as much as one could hold on a nickel, put it in water, and snuff into the nose, or use in a spray. I again thank the gentlemen for their discussion of my paper.

